# Peptide aptamer-based time-resolved fluoroimmunoassay for CHIKV diagnosis

**DOI:** 10.1186/s12985-023-02132-w

**Published:** 2023-07-27

**Authors:** Tonggong Liu, Cheng Gao, Jingzhe Wang, Jianning Song, Xi Chen, Hongfang Chen, Xiaona Zhao, Huanwen Tang, Dayong Gu

**Affiliations:** 1 Department of Laboratory Medicine, Shenzhen Institute of Translational Medicine, Shenzhen Second People’s Hospital, The First Affiliated Hospital of Shenzhen University, 518035 Shenzhen, China; 2 School of Public Health, Dongguan Key Laboratory of Environmental Medicine, Guangdong Medical University, 523808 Guangdong, China; 3 Guangzhou Medical University, 510182 Guangzhou, China; 4 Guangxi University of Chinese Medicine, 530004 Nanning, China

**Keywords:** Chikungunya Virus, Peptide aptamer, Computer-aided design, Molecular docking, TRFIA

## Abstract

**Background:**

Chikungunya virus (CHIKV) and Dengue virus (DENV) have similar clinical symptoms, which often induce misdiagnoses. Therefore, an antigen detection diagnostic system that can clearly identify these two viruses is desirable.

**Methods:**

In this study, we developed a novel peptide with high affinity and specificity to CHIKV, and further constructed peptide aptamer-based TRFIA assay to efficiently detect CHIKV. Peptide aptamer B2 (ITPQSSTTEAEL) and B3 (DTQGSNWI) were obtained through computer-aided design and selected as CHIKV-specific peptide aptamers based on their high binding affinity, strong hydrogen bonding, and RMSD of molecular docking. Then, a sandwich-Time-Resolved Fluoroimmunoassay (TRFIA) was successfully constructed for the detection of the interaction between peptide aptamers and viruses.

**Results:**

When using B2 as the detection element, highly specific detection of CHIKV E2 was achieved with detection limits of 8.5 ng/ml in PBS solution. Variation coefficient between inter-assay showed the disturbances received from the detection of clinical fluid specimens (including serum and urine), were also within acceptable limits. The detection limits for 10-fold dilution serum and urine were 57.8 ng/mL and 147.3 ng/mL, respectively. The fluorescent signal intensity exhibited a good linear correlation with E2 protein concentration in the range of 0-1000 ng/mL, indicating the potential for quantitative detection of E2 protein.

**Conclusions:**

These results demonstrate that the construction of peptide aptamers with high affinity and specificity provides an excellent method for rapid diagnostic element screening, and the developed peptide aptamer B2 contributed to better detection of CHIKV viral particles compared to traditional antibodies.

**Supplementary Information:**

The online version contains supplementary material available at 10.1186/s12985-023-02132-w.

## Introduction

Chikungunya virus (CHIKV) is a type of mosquito-borne virus that is transmitted by the Aedes aegypti and Aedes albopictus mosquitoes. It always causes Chikungunya fever (CHIKF), which is characterized by fever, sudden severe joint pain, and skin rashes [1]. CHIKV was first identified as a rapidly spreading disease at Makonde Plateau in 1953, and the clinical symptoms were similar to dengue fever [2]. This virus was limited to smaller outbreaks in Africa and Asia for the next 50 years [3]. However, in 2005–2006, a CHIKF pandemic occurred on the Indian Ocean island of Reunion, where more than a third of the population became infected with CHIKV [4]. Genome microevolutionary studies of local CHIKV isolates suggested that the outbreak was mainly caused by an amino acid mutation in the CHIKV envelope protein E1-A226V. This mutation enhanced virus replication in insect cells and increased vectorial transmission capacity, which facilitated the spread of the virus [5]. Subsequently, the infection spread globally, because infected travelers from the local area departed to many countries in Europe, Asia, and the Americas, causing local transmission in these countries [6, 7]. The increase in air travel, transmission due to adaptive mutations, lack of effective vaccines, and inadequate mosquito population control measures made CHIKV become a serious global public health problem.

CHIKV belongs to the Togaviridae family of RNA viruses with a 60–70 nm diameter icosahedral nucleocapsid enveloped in a lipid envelope. Two open reading frames (ORFs) are contained in the 11.8 kb positive-sense single-stranded RNA genome [8]. The 5’-terminal ORF codes for a pre-protein that is processed to yield four non-structural proteins including nsP1, nsP2, nsP3, and nsP4. Non-structural proteins are involved in various enzymatic activities that participate in virus particle replication and budding and are important therapeutic targets for drug screening [9, 10]. The 3’-terminal ORF encodes a precursor polyprotein C-pE2-6 K-E1, which is processed to yield five structural proteins including two envelope proteins (E1 and E2) and accessory peptide E3. The envelope protein E2 folds with the help of the accessory peptide E3 and then forms a heterodimer with envelope protein E1, which assembles into a trimeric spike on the virus surface. Mature CHIKV particles consist of 240 E1 and E2 protein monomers [11]. E1 protein mediates the attachment of the virus membrane to the cell membrane, while virus-host cell interaction is mediated by the E2 glycoprotein, which binds to attachment sites, including glycosaminoglycans (GAGs) and the Mxra8 receptor [12]. The structural dominance, conservation of sequences, and high copy numbers of E2 make it become an effective immune target with good potential for immunological detection.

In general, clinical symptoms of mosquito-borne viruses, especially CHIKV, are highly similar to those of Zika and DENV, leading to misdiagnosis easily. CHIKV and DENV have the same endemicity in the tropical and subtropical regions of the Western Hemisphere, and co-infection in mosquitoes has been reported. Thus, it is full of challenging to quickly diagnose CHIKV, which often results in the underestimation of CHIKV infections [13]. Currently, the diagnostic methods for CHIKV mainly include virus culture, virus genome detection, and serological testing. The culture of the virus is considered the golden standard but it is often time-consuming and unsuitable for clinical use [14]. Fluorescent quantitative PCR (qRT-PCR) analysis has become a commonly used clinical detection method. However, it also faces problems such as difficult extraction, long processing time, and high environmental personnel requirements [15]. Then, serological testing is used to detect CHIKV antibodies and serves as a more accurate and complementary detection method after the acute phase of CHIKV infection [16]. Nevertheless, targeting antigens utilized for immunoassay is limited by antibody development, including long animal immunization time, high cost, large inter-batch variability, poor immunogenicity, and low stability [17]. Herein, new tools are desirable to be developed to enhance the sensitivity and specificity of CHIKV diagnosis. As an effective alternative to antibodies, aptamers exhibit many advantages and have been applied in the detection of arboviral antigens. For example, Alexander Bosak et al. used aptamer-gold nanoparticle conjugates to detect Aedes aegypti saliva protein for assessing Zika virus infection status in mosquitoes and Do Thi Hoang Kim et al. constructed a novel immunological assay based on peptide aptamers for detecting Zika virus in clinical specimens [18]. Therefore, it is promising to develop an aptamer-based immunodiagnostic approach for efficient CHIKV antigen-specific diagnostic testing.

In this study, we adopted a computer-aided design process to screen CHIKV E2-specic peptide aptamers. Molecular docking was performed between peptide aptamer and CHIKV E2 to describe their binding mode. A time-resolved fluorescence immunoassay (TRFIA) based on peptide aptamers was constructed to detect CHIKV E2 protein in various buffer systems and the clinical diagnostic features of this method were also evaluated.

## Materials and methods

### Reagents

Europium (III) nanoparticles (0.2 μm in diameter) of carboxylate-coated polystyrene beads (Eu+/PS-COOH Nps) were purchased from Bangs (USA). All peptide aptamers were synthesized by GenScript (China). Recombinant Dengue virus type 2 Envelope Protein (Sino Biological, Cat No.: 40,471-V08B); Recombinant Zika virus Envelope Protein (Sino Biological, Cat No.: 40,543-V08B4); Recombinant Chikv virus Envelope2 Protein (Sino Biological, Cat No.: 40,440-V08B); Anti-DENV E Rabbit Polyclonal Antibody (Sino Biological, Cat No.: 40,471-T62); Anti-CHIKV E Mouse monoclonal Antibody (Sino Biological, Cat No.: M144) were ordered from Sino Biological (China). Anti-Envelope Rabbit Polyclonal (PROSPEC, Cat No.: anti-659) and Antibody Anti- Envelope Mouse monoclonal Antibody (PROSPEC, Cat No.: anti-143) were ordered from PROSPEC (Israel). Chemical reagents such as EDC, Sulfo-NHS, and microplate were obtained from Thermo (China). Bioconjugated reagents, including MES buffer (pH 5.5), PB buffer (pH 8.0), Carbonate-bicarbonate buffer (pH 9.6) and others, were purchased from Sigma-Aldrich (USA). The 96-well Nunc immobilizer amino plates (black) for protein coating were acquired from Thermo (China). Rabbit anti-Mouse IgG Polyclonal Antibody (Cat No.: SPA231) and Mouse Anti Rabbit IgG monoclonal antibody (Cat No.: K20004M) were acquired from Solarbio (China). anti-659: Anti-CHIKV E Rabbit Polyclonal Antibody; M144: Anti-CHIKV E Mouse monoclonal Antibody; anti-143: Anti-CHIKV E Mouse monoclonal Antibody.

### Peptide design and docking studies

Crystal structures of antigenic proteins, including Dengue virus type 2 (DENV-2, PBD ID: 4UTC; resolution: 3.08 Å), Zika virus (ZIKV, PBD ID: 5JHM; resolution: 2.00 Å), and Chikungunya virus (CHIKV, PBD ID: 3N40; resolution: 2.17 Å), were obtained from the Protein Data Bank (PDB). Peptide aptamer subsets of CHIKV envelope protein E were predicted using the three different prediction software, including ABCpred, BCPreds and IEDB-BepiPred [19, 20]. Other peptide aptamers were designed by predicting the sequence of the complementarity-determining region (CDR) of the broadly neutralizing human antibody EDE1 (PBD ID: 4UTC; resolution: 3.0 Å) by using the prediction tool Abnum [21, 22]. 3D structures of the antigen and peptide aptamers were produced after predicting their binding capacity in Autodock Vina software [23]. The mean binding energy of -6.5 kcal/mol was selected as the cut-off value for peptide aptamers, indicating a higher sensitivity for CHIKV and less sensitivity for DENV and Zika. Interaction sites and hydrogen bonds between peptide aptamers and antigens were analysed using PyMOL [24].

### Production of Europium-conjugated peptide aptamer

To produce Europium-conjugated peptide aptamer, 1 mg of lyophilized peptide aptamer powder was dissolved in 1 mL of DMSO solvent and stored at -80 °C. Next, 10 µL of Europium (III) nanoparticles (0.2 μm in diameter) coated with carboxylate-polystyrene beads (Eu+/PS-COOH NPs) were cleaned in MES buffer (0.05 M; pH 5.5) containing 0.02% Tween-20. The washed microspheres were centrifuged at 27,237 g for 10 min, and the supernatants were dropped. Microspheres were dispersed or sonicated, and then 10 µL of EDC (40 mM in MES buffer) and 10 µL of sulfo-NHS (100 mM in MES buffer) were mixed into 180 µL of dispersed microspheres. The reaction mixture was rotated at ambient temperature for over 30 min and then cleaned with 0.1 M phosphate buffer (pH 8.0). Next, 6 µL of peptide aptamer (1 mg/mL) was added to 400 µL of microsphere dispersion, mixed by rotation, and protected from light at room temperature for 1.5 h. The NPs-peptide aptamer was washed twice with phosphate buffer and blocked with 1 mL of Tris-HCL blocking solution containing 2.45 µL of ethanolamine (40 mM in Tris-HCL buffer) and 20 mg of bovine serum albumin (2% in Tris-HCL) for 2 h. After blocking, the NPs-peptide aptamer was washed twice with phosphate buffer and stored in 200 µL of PBS with 0.05% Tween-20 and 0.1% BSA. The final product was stored at 4 °C away from light and diluted 100-fold for subsequent testing.

### Peptide aptamer-based time-resolved fluoroimmunoassay (TRFIA)

Recombinant envelope protein was diluted to the desired concentration (0-1000 ng/ml) using a coating buffer. A 96-well plate was filled with 100 µL of diluted protein standards, then incubated at 4 °C for a whole night. The plates were then blocked by 150 L of 5% skim milk powder at room temperature for 2 h after being washed twice with washing buffer. After blocking, the plate was kept dry and preserved at -20 °C for storage. Subsequently. Then, 100 L of NPs-peptide aptamer was applied to every well and kept at 37 °C for 1 h. The wells were then cleaned with washing buffer five times. A Spark 20 M multimode microplate reader with an excitation wavelength of 365 nm and an emission wavelength of 610 nm was used to detect fluorescence (TECAN, Männedorf, Switzerland).

### Peptide aptamer-antibody pairs “sandwich assay

The recombinant proteins in samples were detected using a sandwich assay with peptide aptamer-antibody pairs. 96-well plates were coated with antibodies (1 g/mL) by overnight incubation at 4 °C. Following a three-time washing with washing buffer, the plates were blocked with 150 L of 5% skim milk powder at room temperature for two hours, and rewashed three times. The plates were then filled with various recombinant protein solution concentrations, together with serum and urine samples, and incubated for 1 h. The plates were cleaned three times before being incubated with 100 L of NPs-peptide aptamer for 1 h at 37 °C. The plates were then cleaned five times, and the Spark 20 M multimode microplate reader was used to read the data.

### Peptide aptamer-TRFIA evaluation

Through fold dilution, standard viral recombinant protein solutions were created, and NPS-peptide aptamers were used for the assay. In order to assess the equation using linear regression and construct a standard curve, the correlation coefficient (R2) was employed as a linearity indicator. The NPS-peptide aptamer assay’s stability in the presence of serum and urine specimens was determined by adding viral recombinant protein to normal human serum and urine specimens and counting the fluorescence counts. The limit of detection (LoD) was determined using the David Armbruster et al. approach [25]. Twenty blank concentrations and low-concentration specimens were used to compute the mean (x) and standard deviation (SD). The sensitivity of this method was measured as below: LoB = x̅ _blank_ + 1.645(SD _blank_) and LoD = LoB + 1.645(SD _low concentration sample_). To assess the specificity of the method, dengue virus envelope protein E and Zika virus protein E were used as potential interferences. Three different concentrations of recombinant protein serum and urine samples were prepared, and each concentration was fluorescently counted 12 times using NPS-peptide aptamers. SD and x̅ values were calculated for the same concentrations. By using the formula CV (%) = SD/x 100%, the intra-assay coefficient of variation (CV) was computed.

### Ethical considerations

The study was approved by The First Affiliated Hospital of Shenzhen University, Shenzhen Second People’s Hospital Institutional Review Board. Negative specimens were collected at the Shenzhen Second People’s Hospital. All experiments and methods were carried out in accordance with relevant guidelines and regulations. Envelope proteins were added to negative human specimens to assess the performance of the assay.

### Statistical analysis

Statistical analysis and graphing had all been conducted, which use GraphPad Prism version 9.0 and Adobe Illustrator 2021. Standard deviations (SD) and sample means (x) are used to express all data. To determine the statistical significance of the differences between the groups, the unpaired t-test, one-way ANOVA, and two-way ANOVA were employed. Values of *P ≤ 0.05, **P ≤ 0.01 and ***P ≤ 0.001 were regarded as statistically significant.

## Results

### Computer-aided design peptide aptamers

**Fig. 1:**
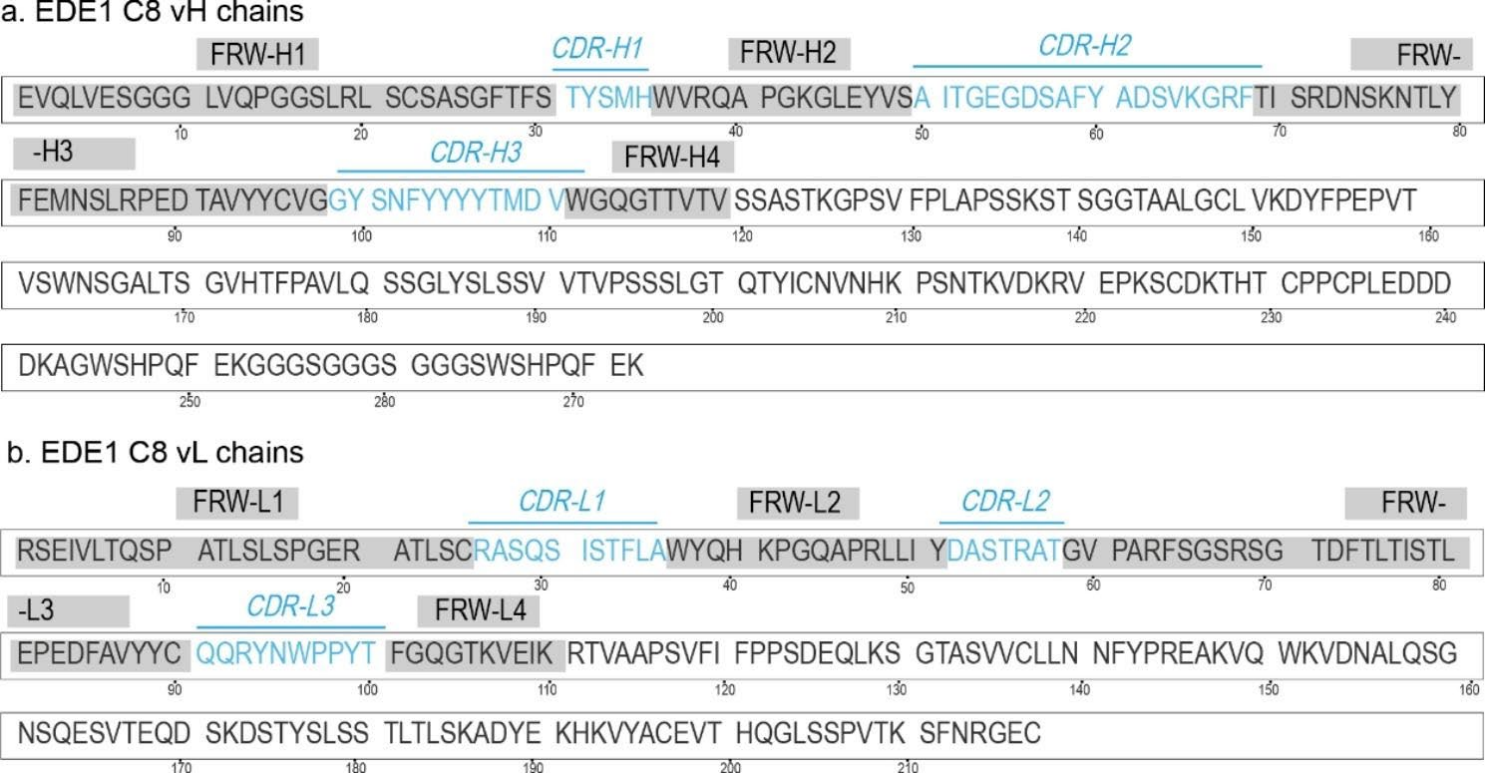
Designing of T1-T5 peptide aptamers. Fab sequences of the broadly neutralizing human antibody EDE1 from the Protein Data Bank (PDB ID: 4UTA; resolution: 3.0 Å). (**a-b**): The complementarity-determining regions (CDRs) and framework regions (FRs) of the heavy and light chains are labelled separately, where CDR-H1 does not meet the requirement for the number of amino acid residues in the peptide aptamer and is removed

Based on structure-based computer-aided design, it helps accelerate the discovery and development of aptamers by minimizing costs and time. In this study, we adopted two different strategies to design structurally specific peptide aptamers. Firstly, we predicted the CDR positions responsible for antigen-antibody interaction in the human broadly neutralizing antibody EDE1 that can bind to the CHIKV envelope protein. The sequence of the heavy and light chain variable regions of the antibody EDE1 was examined using the Abnum tool, and the sequence and position information of the six CDR regions are shown in Fig. 1. SAbPred tool was used as a supplement to the CDR prediction tool. The six CDR positions obtained by the two tools showed the same sequence, with five CDRs(T1-T5) having more than five amino acid residues, meeting the requirements for peptide aptamers. We compared the amino acid sequences of the envelope proteins of three viruses, DENV, ZIKV and CHIKV, and the results are shown in Figure S1. Then three tools were used to predict the linear epitopes of CHIKV, including ABCpred designed 13 epitopes with a threshold greater than 0.7, IEDB-BepiPred designed 13 epitopes with a specificity greater than 0.75, and BCPreds designed 20 epitopes with a threshold greater than 0.7. During the alignment of these epitope sequences, 16 possible repeated epitopes(B1-B16) were obtained, as shown in Table 1. Therefore, only 21 sequences were selected for subsequent molecular docking screening, and the physicochemical properties of these peptide aptamers were analyzed. The sequence and result information are summarized in Table S1.

**Table 1:** Different tools for predicting B-cell epitopes

Tools	No	Position	Epitope	Score	Repetetion	Name of Sequence
	1	40	TLDFELIKTEAK	0.72	TEAK	
	2	55	TLRKYCIEAKLT	0.76		
	3	75	PTQGEPSLNEEQ	0.73	PTQGEPSLNEEQ	B1
	4	93	KHSMVDRGWGNG	0.74		
ABCpred	5	106	GLFGKGGIVTCA	0.78		
	6	117	AKFTCKKNMEGK	0.84		
	7	168	SSTTEAELTGYG	0.80	SSTTEAEL	B6
	8	180	TVTMECSPRTGL	0.82		
	9	198	LLQMEDKAWLVH	0.76		
	10	225	DTQGSNWIQKET	0.70	DTQGSNWI	B3
	11	239	TFKNPHAKKQDV	0.74	FKNPHAKKQDV	B4
	12	273	SSGNLLFTGHLK	0.83		
	13	295	KGMSYSMCTGKF	0.80		
	1	14	GVSGG	0.70		
	2	36	KNKPT	0.69	KNKP	
	3	49	EAKQPA	0.94	EAKQPA	B7
	4	66	TNTTTESRCPTQGEPSLNEEQ	1.37	TNTTTESR/PTQGEPSLNEEQ	
	5	144	HSGEEHAVGNDTGKHGKE	1.21		
IEDB-BepiPred	6	163	KITPQSSTTEAELT	1.11	ITPQSSTTEAEL	B2
	7	221	LPGADTQGSNWI	1.16	TQGSNWI	B8
	8	243	PHAKK	0.96	PHAKK	
	9	326	YEGDGSPCK	1.19	YEGDGSPCK	B9
	10	358	VTEKDSPVNIEAEPPFG	1.23	VTEKDSPV/IEAEPPFG	B10/B11
	11	396	ESRGPFEGKPIPN	1.25	RGPFEGKPIPN	B5
	12	128	KIVQPEN	0.52		
	13	184	ECSPRTGL	0.58	RTG	
BCPred	1	30	VTTMAKNKPTLD	0.71		
	2	44	LIKTEAKQPATL	0.79	LIKTEAK	B12
	3	61	EAKLTNTTTESR	0.96	EAKLT	
	4	74	PTQGEPSLNEEQ	0.67	PTQGEPSLNEEQ	B1
	5	99	GWGNGCGLFGGG	1.00	GWGNG	
	6	146	HAVDTGKHGKEI	0.60		
	7	159	ITPQSSTTEAEL	1.00	SSTTEAEL	B6
	8	176	VTMECSPRTGLD	0.87	VTMECSPRTG	B13
	9	195	QMEDKAWLVHRQ	0.45	QMEDKAWLVH	B14
	10	208	FLDLPWLPWLPG	0.70		
	11	222	TQGSNWIQKETL	0.39	TQGSNWIKET	B15
	12	236	FKNPHAKKQDVV	0.62	FKNPHAKKQDV	B4
	13	249	GSQEGAMHTALT	0.72		
	14	298	MCTGKFKIVKEI	0.78	MCTGKF	B16
	15	322	QYEGDGSPCKIP	0.89		
	16	351	VNPIVTEKDSPV	0.90	VTEKDSPV	B10
	17	364	IEAEPPFGDSYI	0.99		
	18	377	GVEPGQLKLNWL	0.95		
	19	394	RGPFEGKPIPNP	1.00	RGPFEGKPIPN	B5
	20	410	DSTRTGHHHHHH	0.65		

### CHIKV E2-specific peptide aptamer screening by molecular docking

Computer-aided design was a computational method used to simulate the interactions between molecules and protein receptors, to determine the binding modes of a given molecule with a receptor protein and to assess the affinity of the binding [26]. Here, we used a molecular docking approach to calculate whether the designed peptide aptamer could effectively recognize viruses, thereby determining whether the peptide aptamer could be used as a substitute for antibodies in immunodiagnosis.

**Fig. 2:**
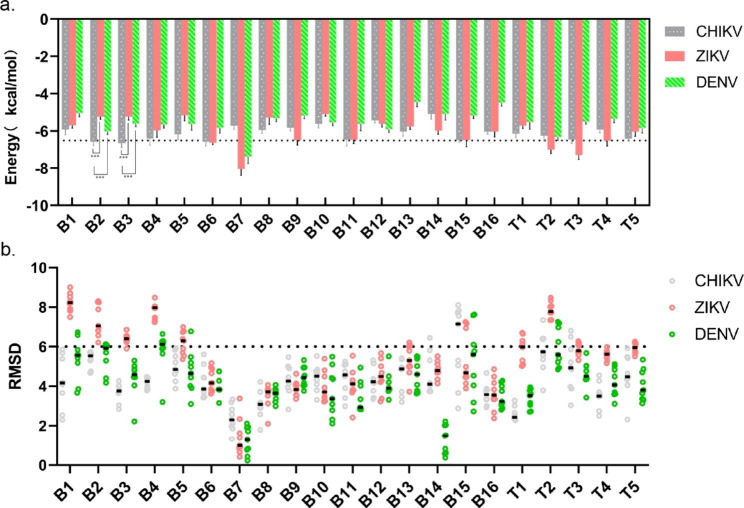
Molecular docking-based screening of CHIKV-specific peptide aptamers (**a**). The binding energy of the 21 peptide aptamers to the three mosquito-borne virus envelope proteins was selected to be below − 6.5 kcal/mol with CHIKV and above − 6.5 kcal/mol with the other two mosquito-borne viruses; *** P < 0.001. (**b**) Root Mean Square Deviation (RMSD) of peptide aptamers docked in conformation with three mosquito-borne virus envelope proteins

Further molecular docking studies were conducted on the 23 peptide aptamers from the initial screening. The crystal structure files of the envelope proteins were obtained from the PDB and used for the virtual screening of these peptide aptamers. The 3D structures of the envelope proteins of the three viruses were visualized using Pymol, as shown in Figure S2. First, the peptide aptamer structure model was predicted using Chem3D and the model was subjected to energy optimization and the peptide aptamer structure model is shown in Figure S3. Auto Dock Vina software was used to dock the peptide aptamers with the envelope proteins of the viruses. As the binding site was unclear, the entire protein molecule was used as the docking receptor, and each peptide aptamers output nine docking complexes. The binding energy and RMSD values were used as parameters for predicting the binding affinity [27]. The RMSD value was calculated by comparing the conformation of the peptide aptamers in the complex with that of its native conformation using Pymol software. The binding energy and RMSD values of the top nine scoring complexes are shown in Table S2. The RMSD cut-off value of 6Å was used as a criterion for the correct prediction of the binding structure. The binding energy average of less than − 6.5 kcal/mol was used as an indicator of strong binding, while binding energies greater than − 6.5 kcal/mol were considered weak, as shown in Fig. 2. Therefore, in order to enhance the understanding and correlation between molecular docking and experimental analysis, we have utilized the molecular docking results to maximize the specificity of peptide aptamers for binding to CHIKV. As shown in Table 2, peptide aptamers B2 (ITQGEPSLNEEQ) and B3 (DTQGSMWI) exhibited strong binding affinity and specificity towards CHIKV, with an average RMSD of less than 6Å. The subsequent experimental analyses were conducted in pH 7.4 phosphate-buffered saline (PBS). Subsequently, the physicochemical properties of peptide aptamers in Table 2, including water solubility and net charge, were analyzed in pH 7.4 phosphate-buffered saline (PBS). Due to the presence of hydrophilic amino acids and large amount of polar amino acids, both peptide aptamers exhibited good water solubility and a slightly negative charge at pH 7.4. contributing to interact with positively charged amino acid residues in the binding pocket of CHIKV.

**Table 2:** Results of peptide aptamer docking results and physicochemical properties (Water. Sol: water solubility. M. W: molecular weight.)

Label	CHIKV		DENV		ZIKV		Net Charge at PH 7.0	Water. Sol	M.W
	Average binding energy(kcal/mol)	Average RMSD(Å)	Average binding energy(kcal/mol)	Average RMSD(Å)	Average binding energy(kcal/mol)	Average RMSD(Å)			
B1	-5.90	4.21	-5.04	5.38	-5.69	8.24	-3.02	good	1328.59
B2	-6.60	3.68	-6.02	5.53	-5.23	7.37	-2.02	good	1275.62
B3	-6.70	4.18	-5.61	4.42	-5.22	6.41	-1.02	good	919.40
B4	-6.40	4.17	-5.66	5.80	-5.97	7.84	2.21	poor	1310.71
B5	-6.17	4.99	-5.63	4.73	-5.16	6.19	0.98	good	1210.65
B6	-6.58	4.17	-5.83	4.01	-6.62	4.35	-2.02	good	836.38
B7	-5.72	2.39	-7.38	1.25	-8.04	1.40	-0.02	good	642.33
B8	-5.94	3.06	-5.32	3.60	-5.28	3.52	-0.02	good	804.38
B9	-5.83	4.31	-5.18	4.47	-6.49	3.90	0.95	good	954.38
B10	-5.62	4.42	-5.54	3.67	-5.10	3.97	-1.02	good	873.44
B11	-6.46	4.40	-5.63	3.43	-6.47	4.06	-2.02	good	858.41
B12	-5.43	4.23	-5.91	4.08	-5.62	4.49	0.98	good	801.50
B13	-6.02	4.53	-4.44	4.58	-5.74	5.31	0.95	good	1079.47
B14	-5.09	4.57	-5.08	1.30	-5.98	4.84	-0.79	good	1255.60
B15	-6.49	6.14	-5.18	5.24	-6.48	5.33	-0.02	good	1162.56
B16	-6.03	3.59	-4.48	3.49	-6.01	3.63	1.94	poor	685.29
T1	-6.14	2.68	-5.52	3.38	-5.71	5.81	-0.02	good	1989.94
T2	-6.26	5.78	-6.33	5.97	-6.99	7.87	3.97	good	1684.68
T3	-6.44	5.01	-5.50	4.62	-7.29	5.87	0.98	poor	1179.62
T4	-5.91	3.54	-5.34	4.17	-6.52	5.58	-0.02	good	720.34
T5	-6.41	4.57	-5.84	4.04	-6.03	5.92	2.97	good	1351.63

### Docking studies

**Fig. 3:**
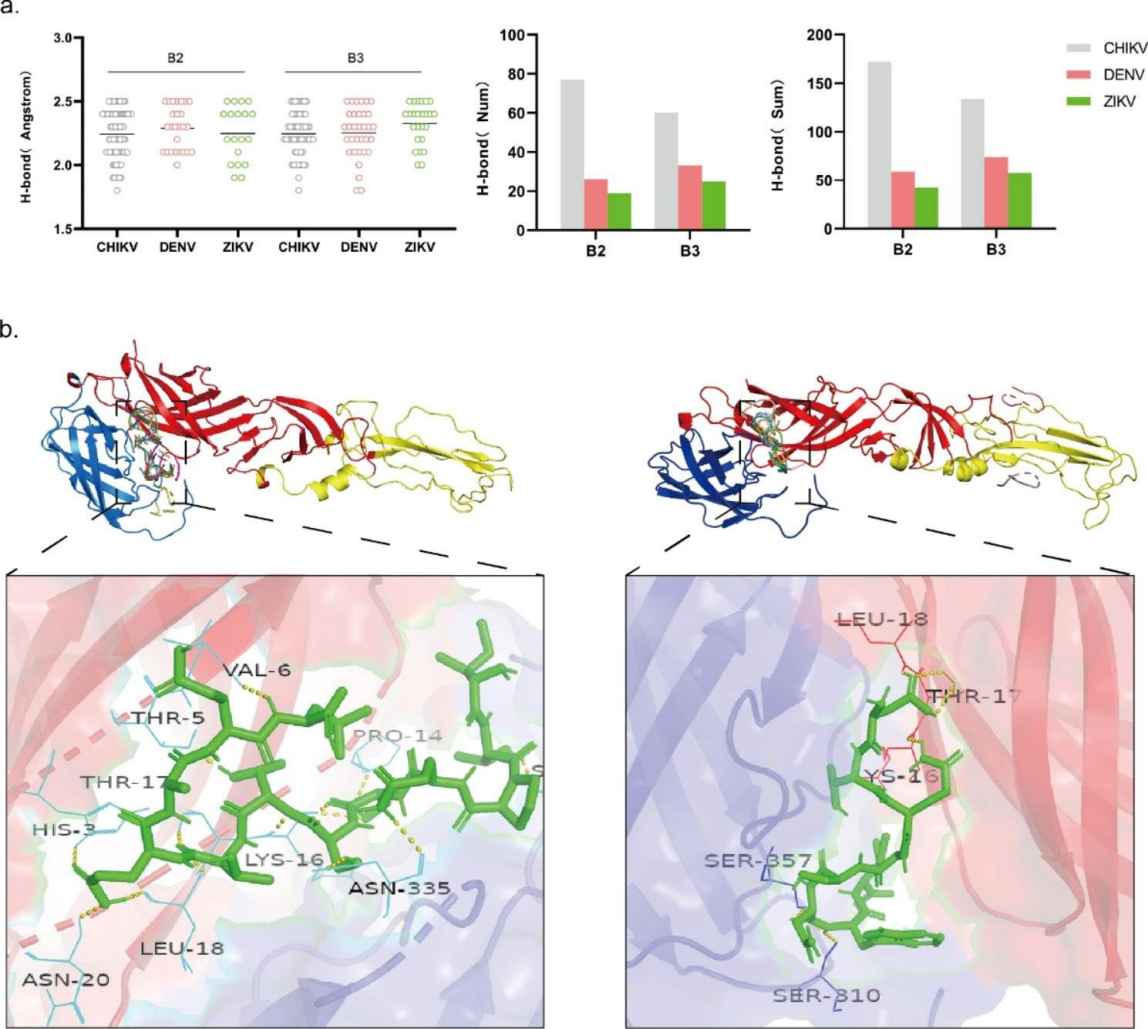
Visualization of peptide aptamer docking results with three mosquito-borne virus envelope proteins and analysis of hydrogen bonding interactions. Using a value of 2.5 Å as an endpoint, Pymol analyses all hydrogen bond (H-bond) lengths, the number of hydrogen bonds, and the sum of lengths resulting from the nine docked conformations (**a**). Examples of the lowest conformations of peptide aptamers B2 (left) and B3 (right) docked to CHIKV binding energy (**b**), red: domainI; yellow: domainII; blue: domainIII; Short yellow sticks: H-bond

The “concave” binding pocket area that contains multiple functional chemical residues is considered to be the ideal binding site in protein-peptide aptamer interactions. These residues interact with the ligand molecule through non-covalent binding [28]. To identify the binding sites and key amino acid residues that regulate the interaction between peptide aptamer and proteins, we analyzed the peptide aptamer-protein complex using Pymol software, with a hydrogen bond cutoff value of 2.5 Å (Fig. 3-a) [29]. For CHIKV, B2 and B3 were expected to have 77 and 60 hydrogen bonds overall, and all hydrogen bonds’ lengths were 172.6 Å and 134.7 Å. As compared to ZIKV and DENV, peptide aptamers and CHIKV produced substantially more and longer hydrogen bonds overall, suggesting a stronger interaction between peptide aptamers and CHIKV compared to DENV and ZIKV. The conformations of peptide aptamers, the envelope proteins of CHIKV, ZIKV, and DENV, and anticipated interaction sites are shown in Fig. 3-b and Figure S4. The pocket between structural domains I and II of the CHIKV envelope protein was anticipated by the majority of models to be its binding site.

### Interaction of peptide aptamers with target proteins

**Fig. 4:**
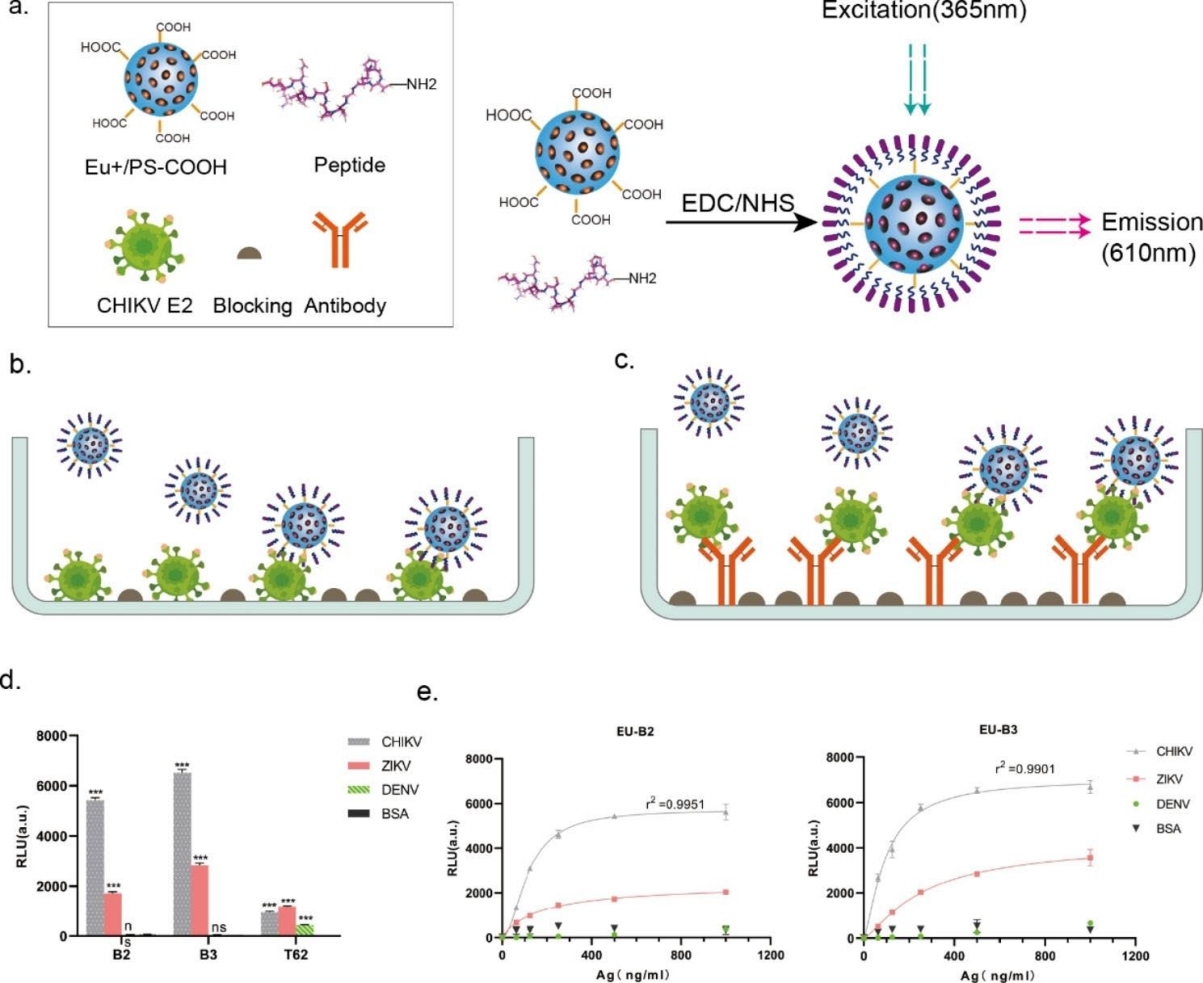
Analysis of direct peptide aptamer-virus interactions. (**a.**) Diagram of covalent coupling of peptide aptamers to europium nanospheres (Eu-Nps). (**b-c.**) Direct and sandwich detection of antigenic protein principles, with analysis of peptide aptamer-antigen interactions by fluorescent signals (excitation: 355 nm and emission:612 nm). (**d**.) 500 ng/mL antigen encapsulated using peptide aptamers and antibodies for direct detection of fluorescent signal intensity; *** P < 0.001. (**e**.) Detection of serially diluted (0-1000 ng/mL) viral recombinant proteins using peptide aptamers

To evaluate the interaction between predicted CHIKV-specific peptide aptamers and target proteins, an excess of peptide aptamers or polyclonal antibody T62 was chemically reacted with Eu+/PS-COOH microspheres through EDC/NHS chemistry to form stable amide bonds (Fig. 4-a). The microsphere surface binding sites were saturated, and the resulting complexes were used as detection elements for TRFIA analysis. Figure 4-b schematically illustrates the direct detection principle of peptide aptamers. Three types of recombinant viral envelope proteins (500 ng/mL) and BSA (negative control) were coated on a 96-well plate. The binding fluorescence value was measured using Eu NPs-conjugated peptides or antibodies at the same concentration. As shown in Fig. 4-a, due to the small molecular weight of the peptide aptamer, more peptide aptamers can be bound by the same mass of fluorescent microspheres as compared to the antibody. Under the same fluorescence microsphere detection conditions, the signal intensities of aptamers B2 and B3 were about four times stronger than those of antibodies. Polyclonal antibody T62 could respond to all three viral proteins simultaneously, while peptide aptamers, as predicted, exhibited stronger signal responses to CHIKV, with weaker fluorescent signals for ZIKV, and neither peptide aptamer detected DENV nor BSA (Fig. 4-d). Based on this, we used peptide aptamers to detect different concentrations of recombinant viral proteins (0-1000 ng/mL), and found that both peptide aptamer B2 (r^2^: 0.9951) and B3 (r^2^: 0.9901) had very high correlation (Fig. 4-e). In addition, we employed various commercially available antibodies to validate the benefits of peptide aptamers binding to the target. To tackle the efficiency concerns linked with direct antibody labeling and enhance the dilution ratio of antibodies in the assay, we implemented an indirect approach for antibody detection. As illustrated in Figure S5, the results demonstrate that the peptide conjugates exhibit signal intensities during antigen binding that are comparable to those of certain antibodies. Therefore, B2 and B3 may function like antibodies to detect viruses.

### Peptide aptamer-linked sandwich TRFIA

Figure 4-c provides a schematic diagram of the sandwich-TRFIA detection method developed for detecting CHIKV viral antigens. In this study, rabbit polyclonal antibody T62 was coated on a 96-well plate for antigen capture in the sandwich method. Eu-peptide aptamers were used to bind to the antigen protein in the buffer system, with the analysis performed by measuring fluorescence intensity. To determine the performance of the sandwich-TRFIA, virus recombinant proteins (0-1000 ng/mL), and BSA protein was employed as a negative control to determine the LoD of the peptide aptamers for CHIKV E2 in the buffer system. According to the fluorescence value, the paired TRFIA of peptide aptamer B2-T62 had an LoD of 8.5 ng/mL for CHIKV E2. In comparison, the paired TRFIA of peptide aptamer B3-T62 had an LoD of 164 ng/mL for CHIKV E. Both peptide aptamers B2 (r^2^: 0.9612) and B3 (r^2^: 0.9602) had good linearity within this concentration range.

### Performance of the peptide aptamer-linked sandwich-TRFIA using human specimens

**Fig. 5:**
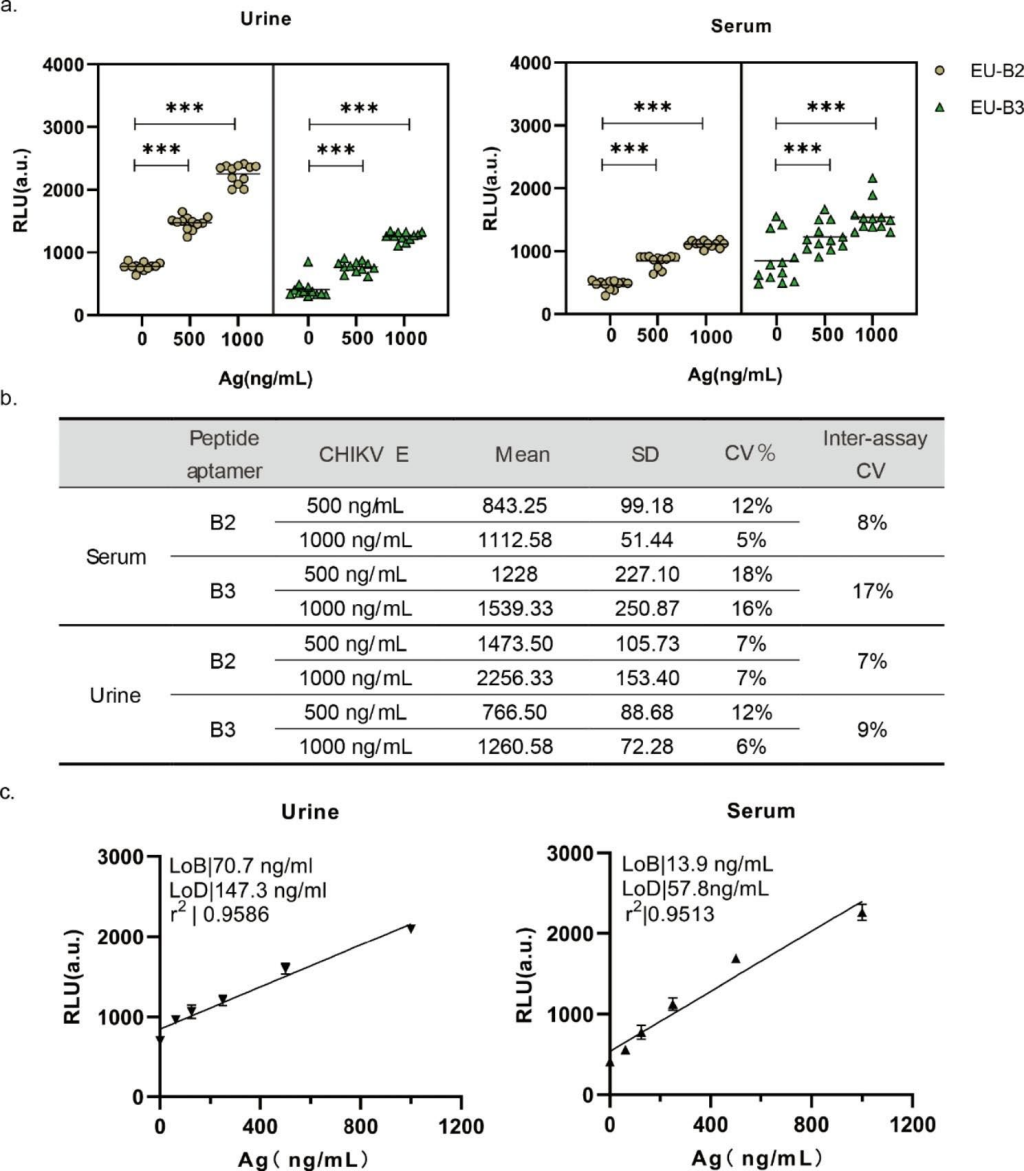
Performance of B2 and B3 as a detection element for CHIKV in fluid specimens. (**a.**) Detection of different concentrations of CHIKV in healthy serum and urine specimens (n = 12); *** P < 0.001. (**b.**) Resistance of peptide aptamers to clinical specimens as measured by the coefficient of variation. (**c.**) Dose-response curves, the limit of detection (LoD), and the limit of blank (LoB) parameters for urine and 10-fold diluted serum

Peptide aptamers’ stability can be significantly decreased by proteases present in serum or plasma samples [30]. Moreover, high concentrations of creatinine and urea can affect normal fluorescence signals [31]. During the acute stage of the illness, samples of urine, serum, saliva, and cerebrospinal fluid from both people and animals have been shown to contain infectious CHIKV [32, 33]. To determine whether the proposed diagnostic system can detect viral particles in human samples, we further evaluated the performance of peptide aptamers in serum and urine samples using sandwich-TRFIA. Two peptide aptamers were tested on human serum and urine samples (n = 12) using a sandwich-TRFIA assay. The antigen protein was diluted to the target concentration in urine and 10-fold diluted serum samples for detection. Figure 5-a demonstrates that the fluorescence signal between negative samples and those with additional antigen protein differed significantly (P < 0.001). Compared to the PBS buffer system, the fluorescence signal from clinical samples was significantly reduced. This reduction may be due to low antibody coating efficiency, lack of clear epitopes for polyclonal antibodies, and interference from serum environments on the fluorescence signal. Meanwhile, the fluorescence signal measured by B2 sandwich-TRFIA showed less variation than B3, suggesting that peptide aptamer B2 may be more suitable for clinical sample detection. Therefore, we calculated the coefficient of variation (CV) for measuring clinical samples using peptide aptamers. The average inter-assay CV for B2 and B3 detection of the two clinical samples was 7.5% and 13% (Fig. 5-b), indicating that peptide aptamer B2 is acceptable since the inter-assay CV is less than 10% [34]. This also suggests that peptide aptamer B2 has less interference from clinical samples and is more promising for clinical sample detection. The analytical sensitivity of peptide aptamer B2 sandwich-TRFIA was determined by diluting virus protein in human fluid samples. As shown in Fig. 5-c, in urine samples (r^2^: 0.9586), the LOB and LOD for peptide aptamer B2 were 70.7 ng/mL and 147.3 ng/mL. In 10-fold diluted serum samples (r^2^: 0.9513), the LOB and LOD for peptide aptamer B2 were 13.9 ng/mL and 57.8 ng/mL. The peptide-aptamer sandwich TRFIA may be a useful tool for identifying viral antigens in patient samples, according to these findings.

## Conclusions

Currently, no approved vaccination or specific therapy for CHIKV infection, which is a global public health problem due to the adaptive mutations of CHIKV that can increase vector transmission capacity and lead to sudden large-scale outbreaks [35]. Accurate differential diagnosis of CHIKV infection is crucial for patient health and epidemic control, especially given the existence of similar clinical symptoms and co-infection with DENV. However, the presence of antibody-dependent enhancement (ADE) in CHIKV infection limits the therapeutic and protective potential of CHIKV antibodies and hinders the development of CHIKV antigen detection methods [36]. Recently, computer-aided design peptide aptamers have gained attention and have been applied to diagnostic platforms. In this study, we attempted to develop a computer-aided designed detection element for CHIKV using peptide aptamers as a substitute for traditional antibodies and construct a corresponding diagnostic method for differential diagnosis of CHIKV.

On the surface of CHIKV icosahedral structure is covered with dimeric units composed of the E1 and E2 proteins. Among them, the E2 protein is highly conserved and immunogenic, making it an ideal molecular target. In this study, we selected different bioinformatics tools to screen for candidate epitope sequences and obtained the CDR sequences of traditional antibodies, which were used as peptide aptamers for further screening. We evaluated the performance of peptide aptamers by molecular docking with envelope proteins of three different viruses and used various criteria to screen for target-specific peptide aptamers. Two hydrophilic CHIKV E2-specific peptide aptamers, B2 (ITPQSSTTEAEL) and B3 (DTQGSNWI) were obtained, demonstrating the potential of this computer-aided designed peptide aptamers generation method to address the issue of antibody scarcity with lower monetary and time costs.

The TRFIA with fluorescence signal detection outperformed ELISA in terms of detection efficiency. Among the most promising fluorescent materials is europium nanoparticles [37], but they have not yet been used in CHIKV diagnostic systems. Interestingly, when we used fluorescence microspheres coupled with peptide aptamers and antibodies, due to the smaller molecular weight of the peptide aptamer (1 kDa), the same amount of fluorescent microspheres could bind more peptide aptamers, and the smaller molecular weight also made it easier for the peptide aptamer to access hidden antigenic sites, thereby enhancing the signal strength of detection [38]. In summary, when used for detection, peptides have lower costs and stronger signal responses than antibodies, which is beneficial for the development of diagnostic systems. We then developed a sandwich-TRFIA detection method using peptide aptamer-antibody pairs and applied it to detect CHIKV, distinguishing it from other mosquito-borne viruses (DENV and ZIKV). The detection limits of peptide aptamers B2 and B3 for CHIKV were 8.5 ng/mL and 164 ng/mL, in a PBS buffer system.

However, the stability of peptide aptamers is limited in bodily fluid specimens such as serum, as the complex composition of bodily fluids may interfere with the peptide-antigen interaction. In our study, background fluorescence was influenced by bodily fluid samples, resulting in a reduced low-background advantage of europium nanoparticles. Meanwhile, the stability of peptide aptamer B3 was severely affected in serum and urine samples, with an inter-assay CV greater than 10%, while the stability of peptide aptamer B2 performed well in bodily fluid samples. Therefore, peptide aptamer B2 is more suitable for clinical specimen testing. Ultimately, we detected the detection limit in 1:10 diluted serum and urine samples, which were 57.8 and 147.3 ng/mL. The peptide-linked sandwich TRFIA is suitable for screening clinical specimens.

Computer-aided design of peptide aptamers has provided a new strategy for antibody production with lower costs, better uniformity, simple chemical modification, and more binding sites, offering possibilities for replacing antibodies in immunodetection. This approach has the potential to replace antibodies in immunodetection. However, there are several potential limitations to our study. Firstly, we focused solely on simple peptide aptamer structures, while strategies such as cysteine-based cyclic peptides and aromatic amino acid modifications can offer current peptide aptamers stronger stability and more complex structural types, which may further improve their binding performance but require more time for effective modifications [39]. Secondly, in order to establish a standardized CHIKV diagnostic method, it is necessary to optimize the reagents and detection procedures used in this study [40]. Finally, it’s also vital to note that the binding sites and interactions between peptide aptamer-E2 complexes were determined using computer simulations and lack crystallographic parameters from X-ray diffraction (XRD) or cryo-electron microscopy.

## Discussion

In summary, a computer-aided design approach was developed to construct peptide aptamers that meet the specific needs of CHIKV serological diagnosis. In comparison to the traditional immunological method, TRFIA significantly improved the sensitivity in diagnosing CHIKV infection, enabling the differential diagnosis of CHIKV and other mosquito-borne viruses (DENV and ZIKV) in human serum and urine specimens. These results showed the computer-based design of peptide aptamer provided the potential to be an alternative to antibodies for diagnostic applications.

## Electronic supplementary material

Below is the link to the electronic supplementary material.

Supplementary Material 1

Supplementary Material 2

Supplementary Material 3

Supplementary Material 4

Supplementary Material 5

Supplementary Material 6

Supplementary Material 7

Supplementary Material 8

## Data Availability

The data that support the findings of this study are available from the corresponding author upon reasonable request.
