# Supplementary figures and images for: Peptide aptamer-based time-resolved fluoroimmunoassay for CHIKV diagnosis
Source: Virol J. 2023 Jul 27;20:166. doi: 10.1186/s12985-023-02132-w (PMC10375649; doi:10.1186/s12985-023-02132-w)

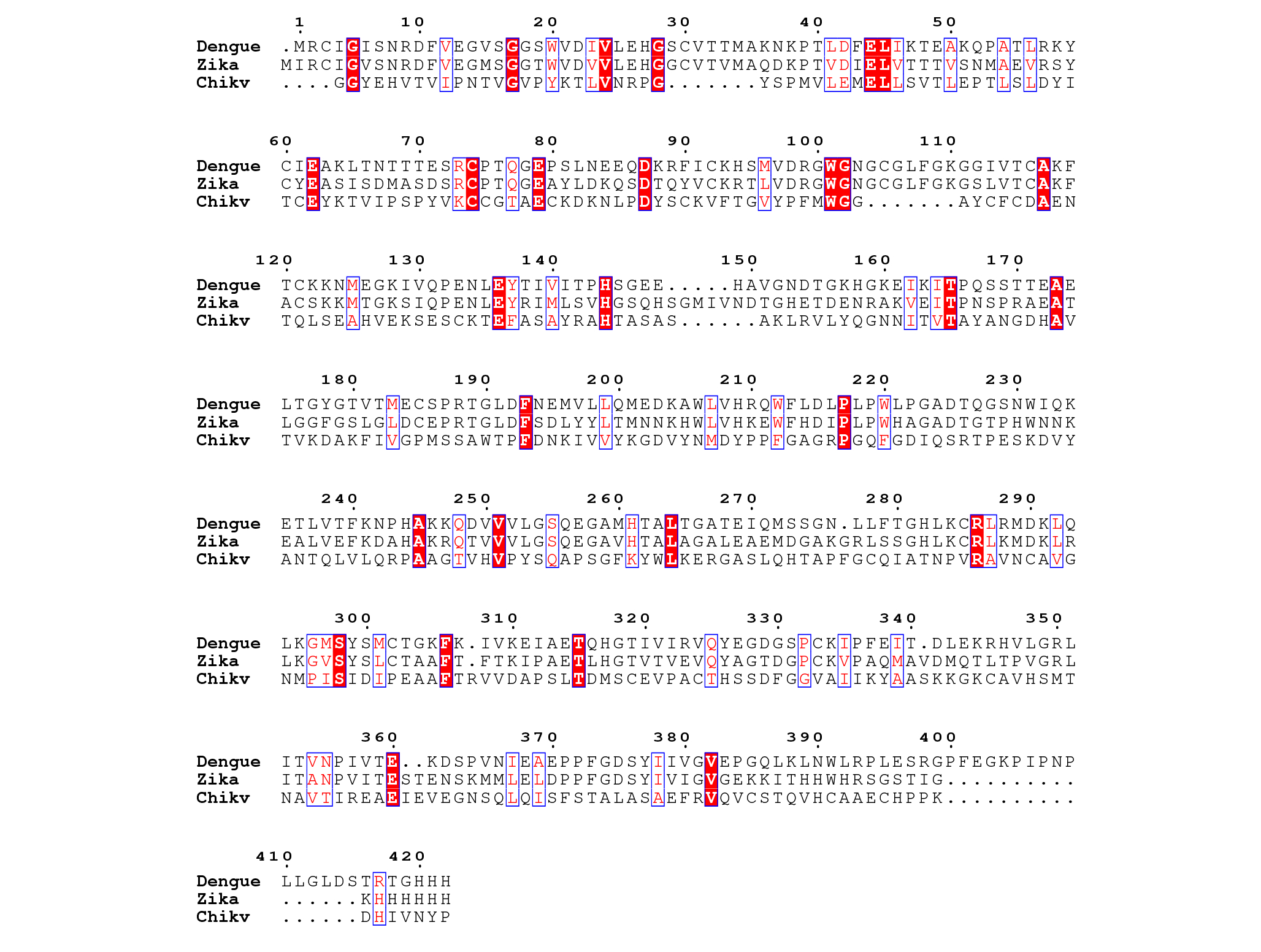

Supplement: Supplementary file 1 — Supplementary Material 1 [file 12985_2023_2132_MOESM1_ESM.png]

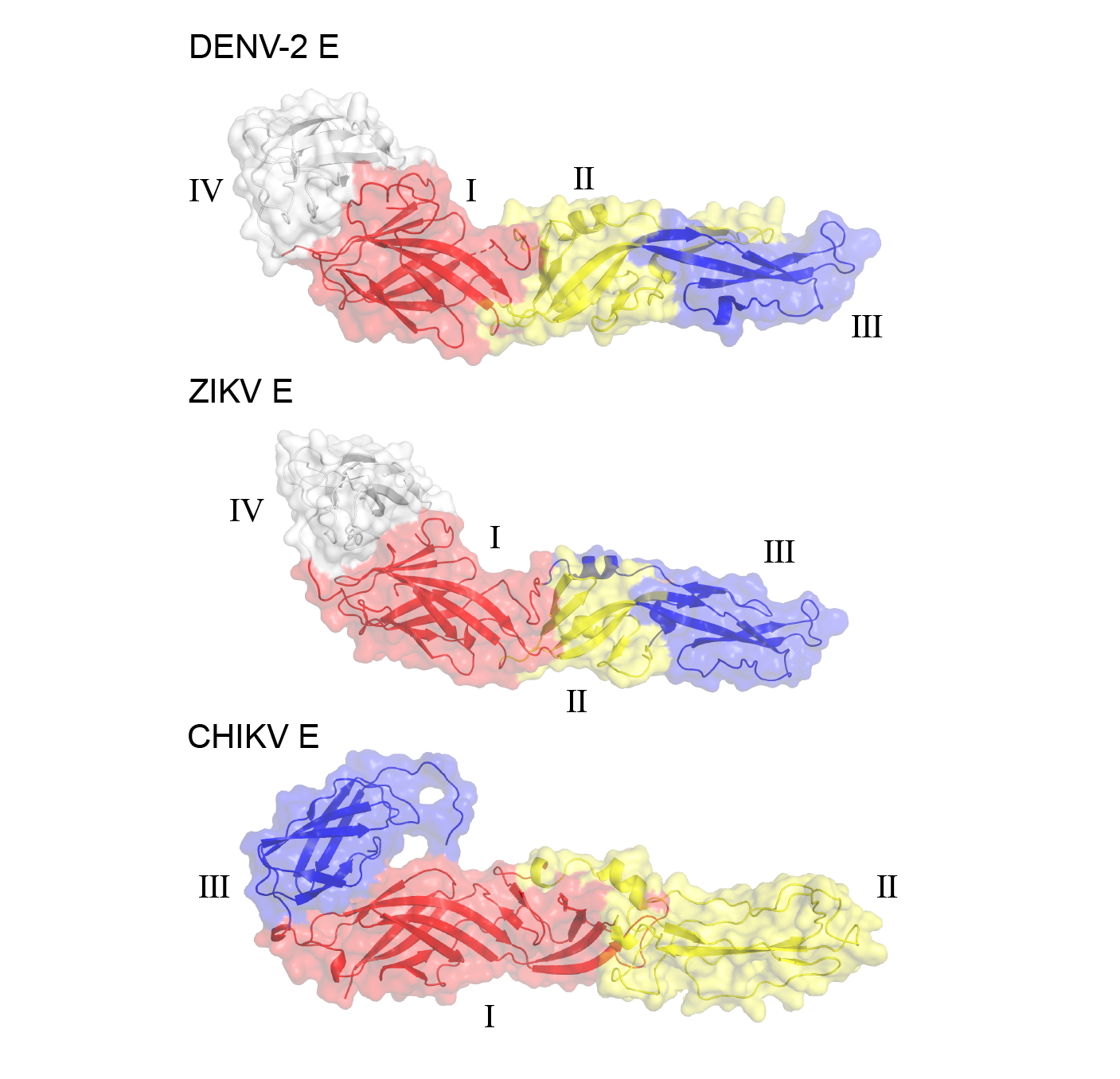

Supplement: Supplementary file 2 — Supplementary Material 2 [file 12985_2023_2132_MOESM2_ESM.png]

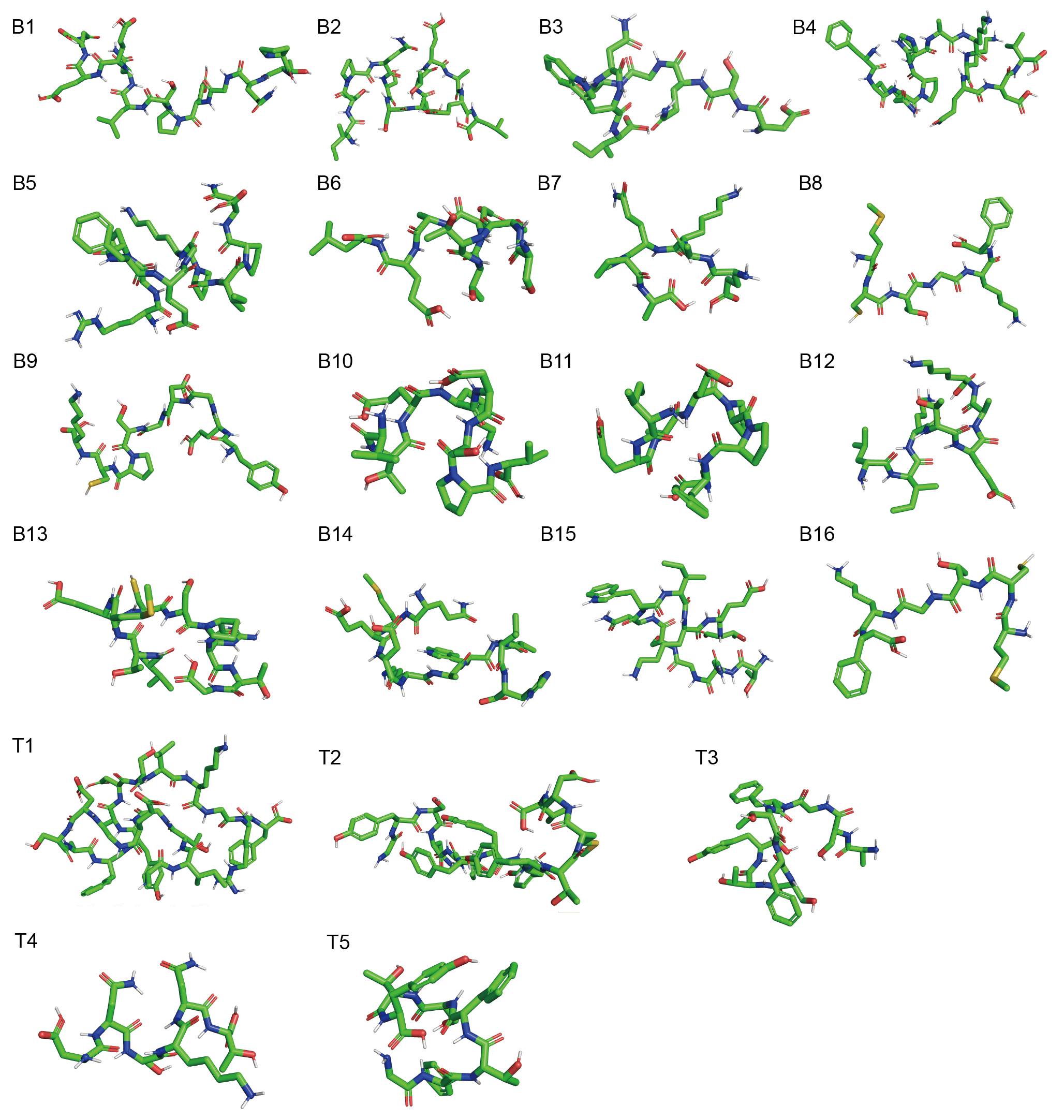

Supplement: Supplementary file 3 — Supplementary Material 3 [file 12985_2023_2132_MOESM3_ESM.png]

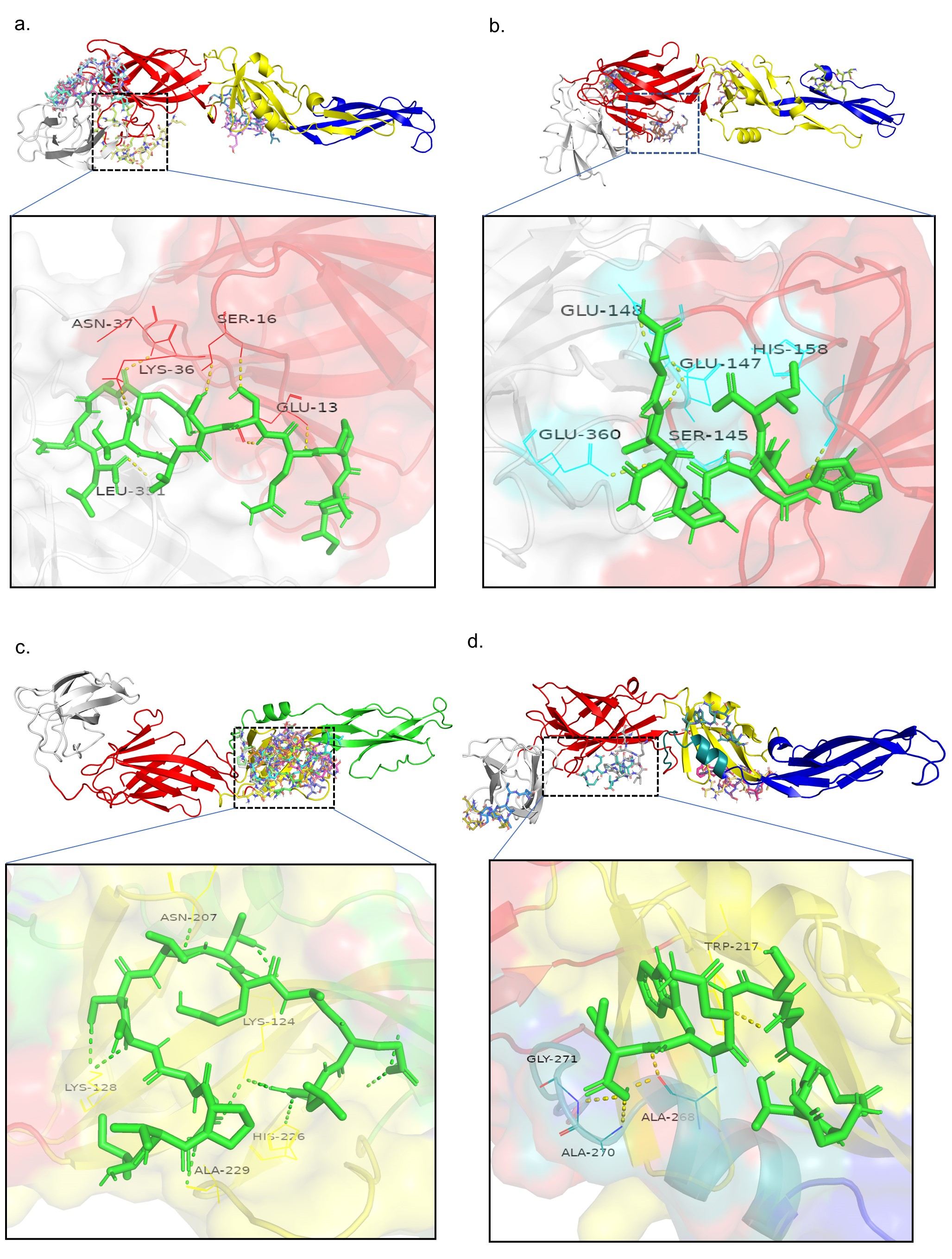

Supplement: Supplementary file 4 — Supplementary Material 4 [file 12985_2023_2132_MOESM4_ESM.png]

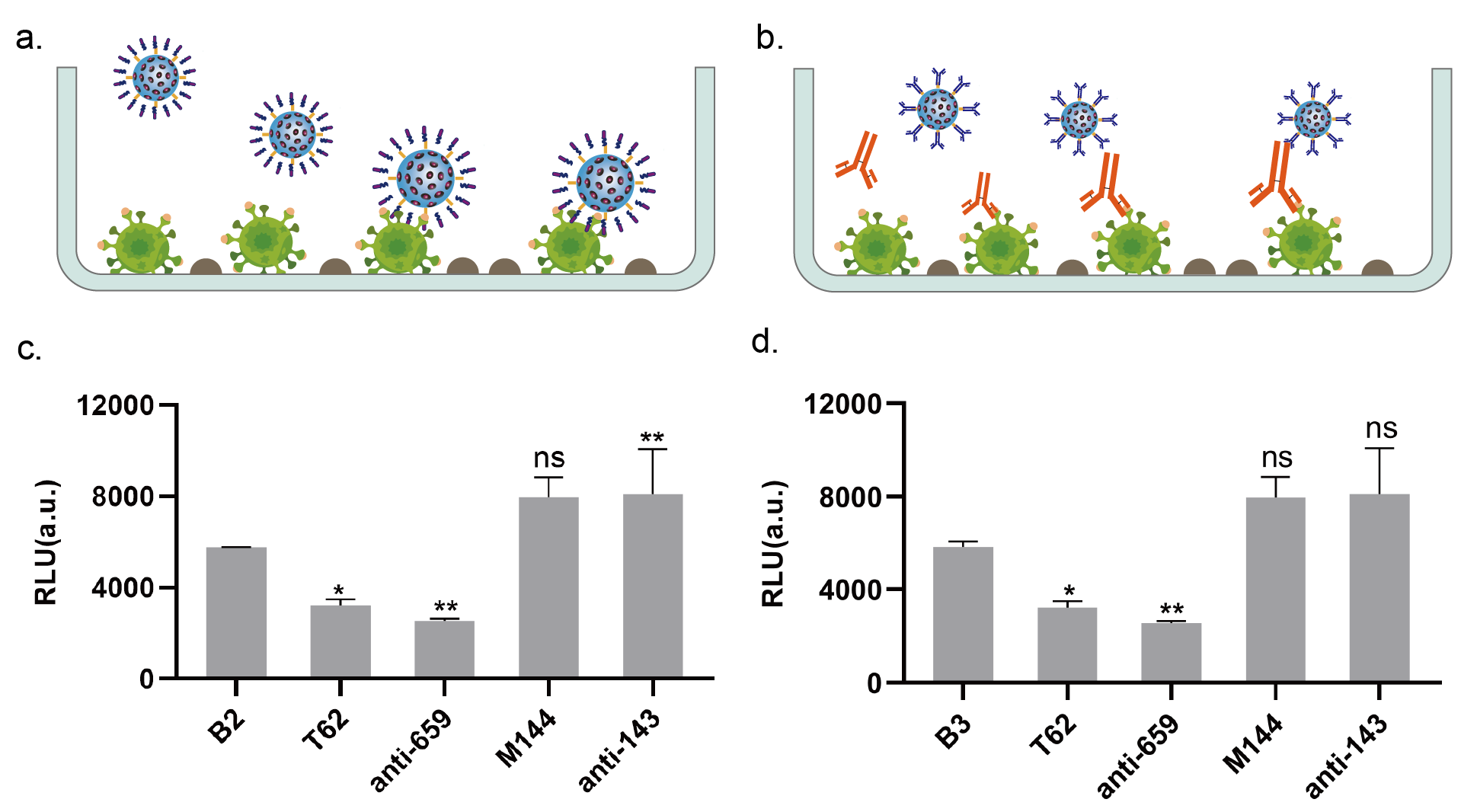

Supplement: Supplementary file 8 — Supplementary Material 8 [file 12985_2023_2132_MOESM8_ESM.png]
