# Supplementary material for: Peptide aptamer-based time-resolved fluoroimmunoassay for CHIKV diagnosis
Source: Virol J. 2023 Jul 27;20:166. doi: 10.1186/s12985-023-02132-w (PMC10375649; doi:10.1186/s12985-023-02132-w)
Supplement: Supplementary file 7 — Supplementary Material 7 [file 12985_2023_2132_MOESM7_ESM.docx]

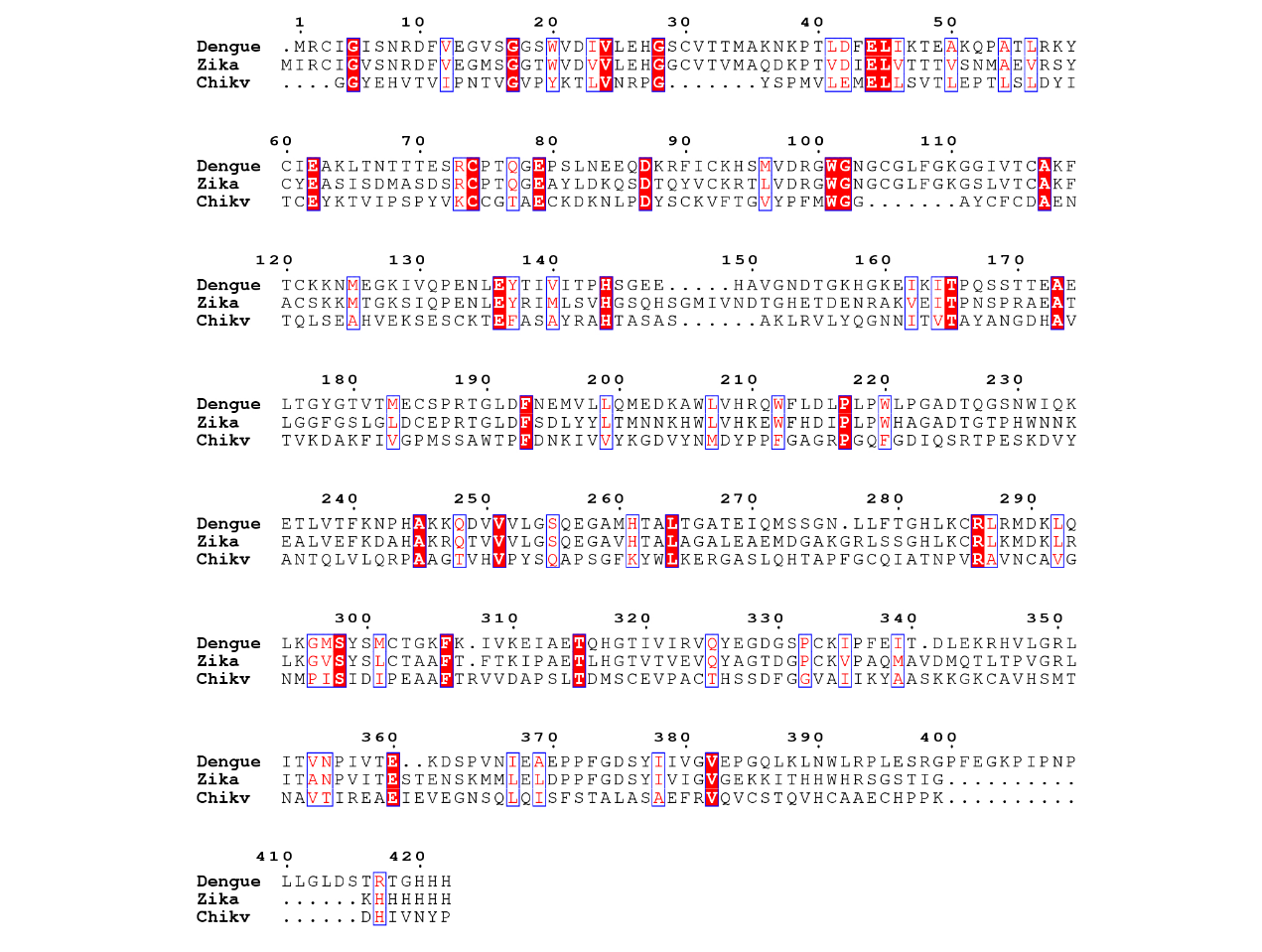


**Figure S1** Sequences of three mosquito-borne virus envelope proteins E (DENV, ZIKV and CHIKV) and their comparative results.


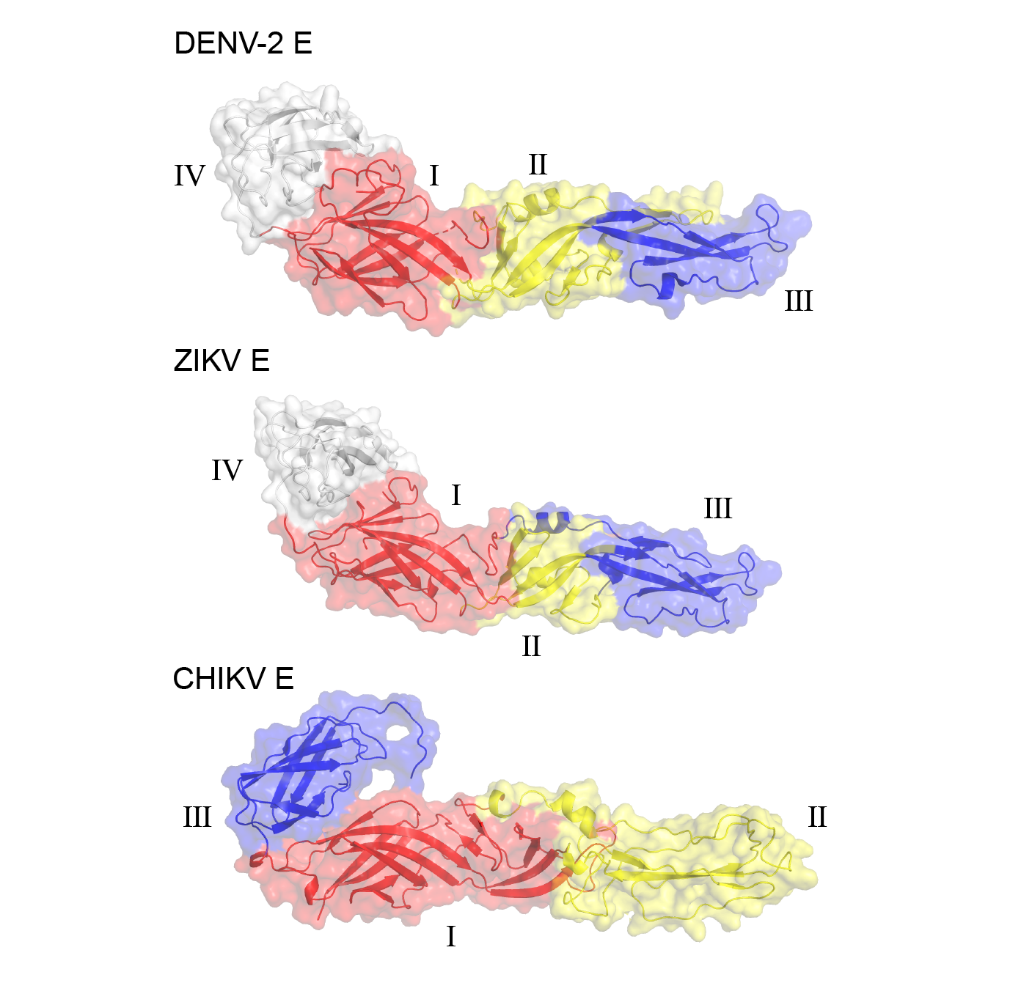


**Figure S2** Crystal-resolved structures of three mosquito-borne virus envelope protein E. Red: domain Ⅰ; yellow: domain Ⅱ; blue: domain Ⅲ; grey: domain Ⅳ.


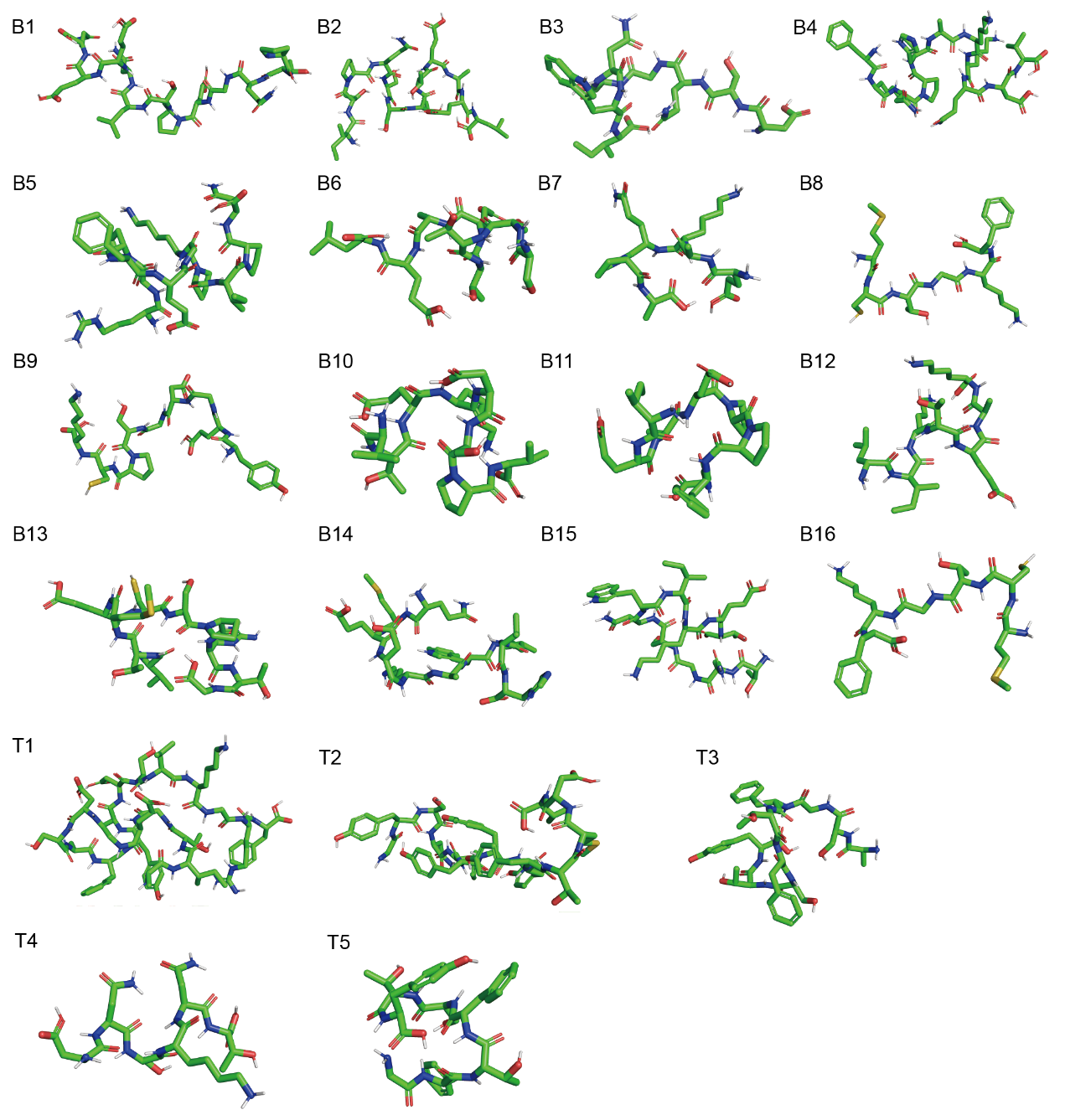


**Figure S3** Predicted structure of 21 peptide aptamers


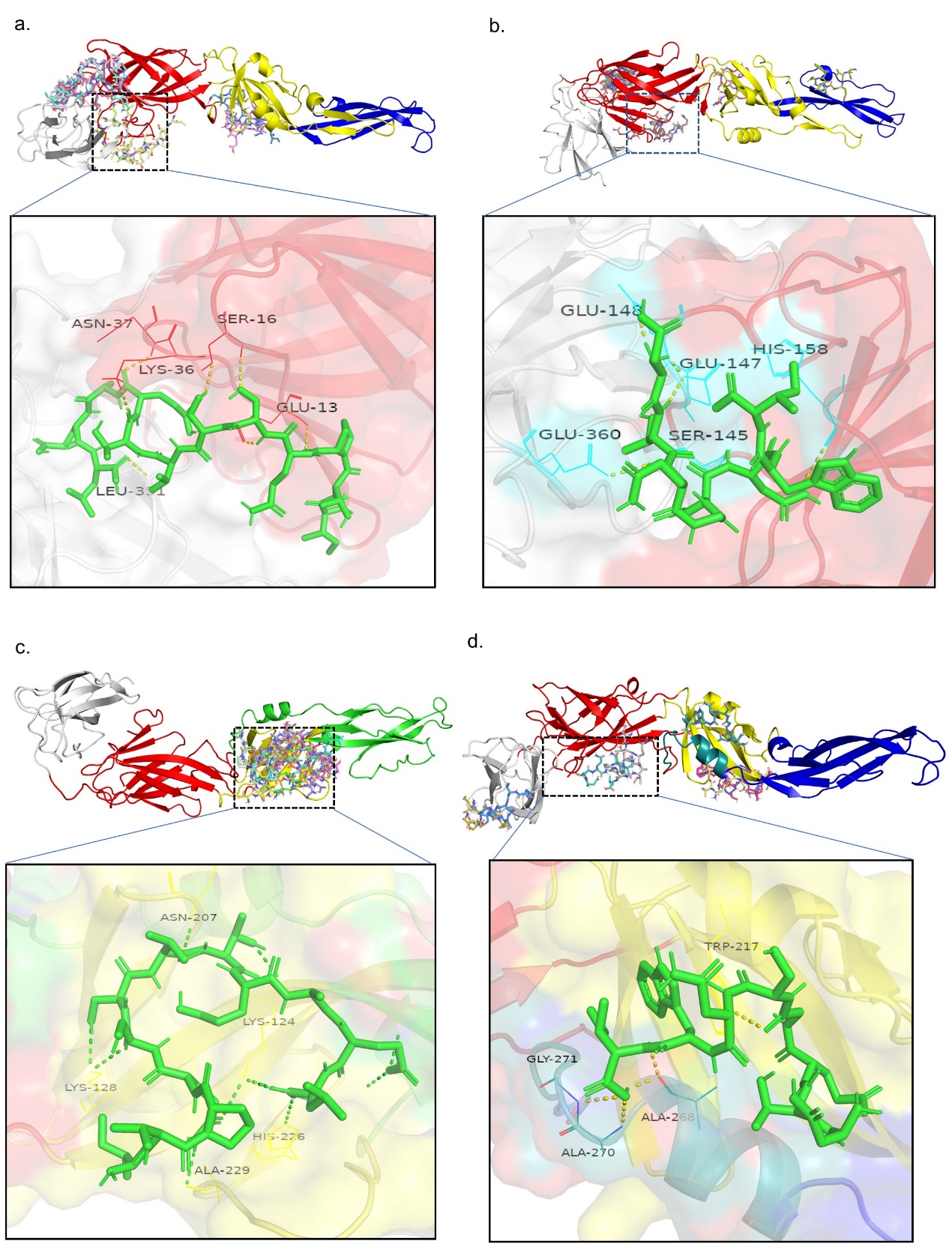


**Figure S4** Diagram of docking of B2 and B3 molecules. (a-b.) The binding sites of B2 and B3 after docking with DENV are shown (top), and the interaction of the already optimal model with the protein (bottom). (c-d.) The binding sites of B2 and B3 after docking with ZIKV are shown (top), and the interaction of the already optimal model with the protein (bottom). Red: domain Ⅰ; yellow: domain Ⅱ; blue: domain Ⅲ; grey: domain Ⅳ, short yellow sticks: H-bond.


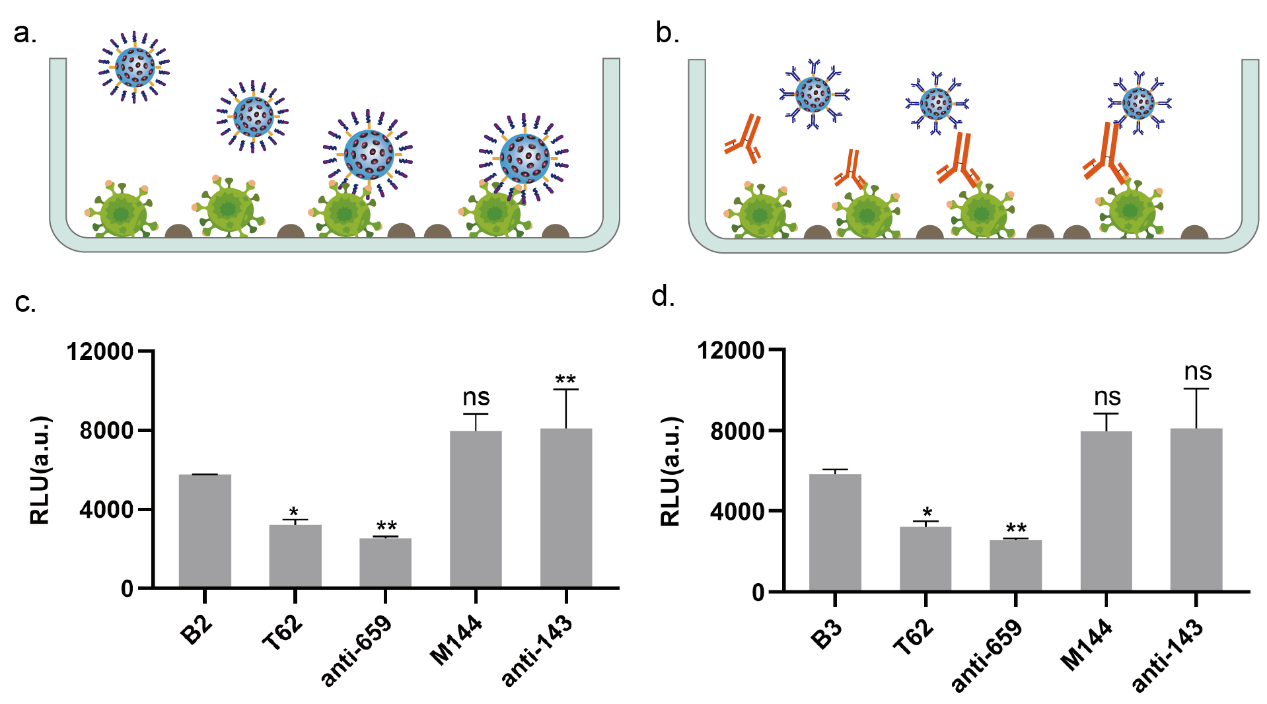


**Figure S5** Comparison of peptide aptamers and antibodies on CHIKV binding. (a-b): Schematic illustration on detection of antigenic proteins by using peptide aptamers and antibodies. Analysis of interactions by fluorescent signals (excitation: 355 nm, emission: 612 nm). (c-d): 500 ng/mL of antigen was incubated with 0.3uM of NPs-peptide aptamer and antibodies, and fluorescence signal intensity was detected by Spark 20M multimode microplate reader. **: P < 0.01, *: P < 0.05, ns: no statistical significance.

**Table S1** Design of peptide aptamer sequences and analysis of their physicochemical properties (GRAVY: grand average of hydropathicity).

**Table S2** Docking results of all peptide aptamers to envelope protein E. (uploaded in a single supplementary file)
